# Supplementary material for: Improving the accuracy of model-based quantitative nuclear magnetic resonance
Source: Magn Reson (Gott). 2020 Jul 2;1(2):141–53. doi: 10.5194/mr-1-141-2020 (PMC10500698; doi:10.5194/mr-1-141-2020)
Supplement: The supplement related to this article is available online at: https://doi.org/10.5194/mr-1-141-2020-supplement. [file mr-1-141-supplement.zip › mr-1-141-2020-supplement-title-page.pdf]

Supplement of Magn. Reson., 1, 141–153, 2020
https://doi.org/10.5194/mr-1-141-2020-supplement
© Author(s) 2020. This work is distributed under
the Creative Commons Attribution 4.0 License.

Open Access
MAGNETIC RESONANCE

Supplement of

Improving the accuracy of model-based quantitative nuclear magnetic resonance

Yevgen Matviychuk et al.

Correspondence to: Yevgen Matviychuk (eugene.matviychuk@canterbury.ac.nz)

- mr-1-141-2020-supplement-title-page.pdf
- OrganicMixtures_Benchtop.jdx
- OrganicMixtures_HF.jdx
- Thiamine.jdx

The copyright of individual parts of the supplement might differ from the CC BY 4.0 License.
